# Rodent models of Parkinson's disease: beyond the motor symptomatology

**DOI:** 10.3389/fnbeh.2013.00175

**Published:** 2013-11-26

**Authors:** Filipa L. Campos, Miguel M. Carvalho, Ana C. Cristovão, Goun Je, Graça Baltazar, António J. Salgado, Yoon-Seong Kim, Nuno Sousa

**Affiliations:** ^1^Life and Health Sciences Research Institute (ICVS), School of Health Sciences, University of MinhoBraga, Portugal; ^2^ICVS/3B's, PT Government Associate LaboratoryBraga/Guimarães, Portugal; ^3^Burnett School of Biomedical Sciences, College of Medicine, University of Central FloridaOrlando, FL, USA; ^4^Department of Medical Sciences, CICS-UBI Health Sciences Research Centre, University of Beira InteriorCovilhã, Portugal

**Keywords:** Parkinson's disease, non-motor symptoms, motor deficits, paraquat, α-synuclein, 6-OHDA, rat

## Abstract

Parkinson's disease (PD) is classically characterized by motor symptoms; however, non-motor symptoms (NMS) are increasingly recognized as relevant in disease-state, given the associated alterations in mood (depression and anxiety) and cognition. Here, particularly in regards to NMS, we aimed to compare the motor, emotional and cognitive behavior of three animal models of PD that trigger dopaminergic (DAergic) degeneration on both brain hemispheres: (i) the 6-hydroxydopamine (6-OHDA, 8 or 6 μg) lesion model; (ii) the paraquat (PQ) induced model, and (iii) a genetic model based on α-synuclein overexpression (α-syn). 6-OHDA and α-syn vector were injected bilaterally in the *substantia nigra pars compacta* (SNpc) of adult male Wistar rats; as for PQ delivery, micro-osmotic pumps were implanted in the interscapular region. Motor deficits were observed in all models, with histological analysis of tyrosine hydroxylase positive cells in the SNpc revealing a significant loss of DAergic neurons in all animal models. In addition, the α-syn animal model also presented a reduction in exploratory activity, and the 6-OHDA and PQ animals displayed a significant increase in both depressive- and anxiety-like behavior. Interestingly, cognitive impairment (working memory) was only observed in the 6-OHDA model. Overall, these PD models are suitable for mimicking the motor symptoms associated to PD, with each encompassing other relevant NMS components of the disorder that may prove beneficial for further studies in PD.

## Introduction

Parkinson's disease (PD), one of the most frequent neurodegenerative disorders, is characterized by the progressive loss of dopamine (DA) neurons in the *substantia nigra pars compacta* (SNpc), leading to striatal DA depletion. This degeneration of DAergic nigrostriatal system is largely responsible for the classical motor signs, including: resting tremor, muscle rigidity, and bradykinesia (Fearnley and Lees, 1991; Olanow and Tatton, 1999). Furthermore, non-motor symptoms (NMS) are also becoming increasingly recognized as relevant symptoms in PD patients (Langston, 2006; Chaudhuri and Schapira, 2009); however, these are still frequently undiagnosed and, therefore, left untreated (McDowell and Chesselet, 2012).

Of particular concern, NMS can include emotional and cognitive deficits, sleep disorders, and autonomic, gastrointestinal, and sensory dysfunction (Chaudhuri and Schapira, 2009). In fact, depression is one of the most frequently encountered psychiatric problems in PD patients and can appear early in the progression of the disease (Aarsland et al., 2012). Anxiety may also be a comorbidity for PD patients, affecting up to 40% of individuals, sometimes in association with depression (Walsh and Bennett, 2001; Martinez-Martin and Damian, 2010). Relevant, these neuropsychiatric alterations are often accompanied by cognitive impairments, which can occur in the early stages of the disease. However, of note, this cognitive “dysfunction” is more common in older patients with advanced PD, among whom dementia can affect up to approximately 24–31% of patients (Aarsland et al., 2005; Chaudhuri and Schapira, 2009; Kehagia et al., 2010).

In this context, taking into account the influence of the NMS in the quality of life of PD patients, it is of interest to assess whether NMS can also be studied in animal models of PD. The 6-hydroxydopamine (6-OHDA) rat model has been the most extensively studied animal model of PD, including results from our group indicating that the unilateral 6-OHDA-lesion in the medial forebrain bundle is a suitable model to investigate depressive-like behavior and exploratory activity impairments associated with PD (Carvalho et al., 2013). Usually 6-OHDA is administrated unilaterally, leading to an asymmetric motor behavior that can be quantified and correlated with the degree of lesion; however, bilateral models mimic more closely the condition in humans (Deumens et al., 2002). Few studies have used bilateral injections of 6-OHDA in the striatum, and the available results are quite conflicting. For instance, while one study showed that injured animals presented a depressive-like state, a decrease in anxiety-like behavior and alterations in social behavior without changes in cognitive functions (Branchi et al., 2008), another study reported that a similar lesion led to the development of cognitive deficits as well as a depressive and anxious-like state (Tadaiesky et al., 2008). There are also reports indicating that rats injected bilaterally in the SNpc also present alterations in depressive-like behavior and in cognitive performance tasks (Ferro et al., 2005; Santiago et al., 2010).

Furthermore, as several epidemiological studies have reported that exposure to environmental toxins, such as herbicides, results in an increased risk of PD (McCormack et al., 2002), studies in rodents have also been performed to assess if herbicides, such as paraquat (PQ), rotenone, and maneb, are able to reproduce classical features of the disease. However, the ability of these models to mimic NMS has been less explored and to the best of our knowledge there is only one report showing that mice treated with PQ develop an anxiety-like state (Litteljohn et al., 2008). Again, the possibility of using this model to study NMS of PD remains to be determined.

Finally, genetic models of PD are more recent and have been largely focused on overexpression of wild-type (wt) or mutated α-synuclein (α-syn) in transgenic mice. In spite of providing a suitable model to study the involvement of α-syn in PD pathogenesis, this model present several limitations. In fact, none of the transgenic α-syn mouse models presented the key features of the disease, namely the extensive nigrostriatal DAergic degeneration or the motor impairments (Magen and Chesselet, 2010). An alternative to these transgenic models is the use of viral vectors to promote the overexpression of α-syn specifically in the nigrostriatal system. The ability of such approach to reproduce many features of the disease, such as the progressive and severe degeneration of the nigrostriatal pathway and motor abnormalities, has proved successful in some studies (Kirik et al., 2002; Decressac et al., 2012a,b); yet, NMS have never been investigated in this model.

The aim of the current study was to use three different rat models of PD (6-OHDA, PQ administration, and adeno-associated virus (AAV)-mediated overexpression of α-syn) to assess emotional and cognitive behaviors, in parallel with motor performance. This phenotypic characterization was complemented with the immunohistological evaluation of the nigrostriatal lesion.

## Materials and methods

### Animals and experimental protocol

Experiments were conducted in accordance with the national ethical requirements for animal research, and with the European Convention for the Protection of Vertebrate Animals Used for Experimental and Other Scientific Purposes. Young adult male Wistar rats (Charles River, Barcelona, Spain) weighing 300–350 g at the beginning of the experiments, were housed in pairs, in appropriate cages, under standard controlled conditions (12 h light/12 h dark cycle; room temperature 22°C; food and water freely available). After surgeries, the animals were allowed to recover for 5 weeks before starting the behavioral assessment. Starting in the sixth week after surgery, all animals were exposed to the following sequential behavioral tests: elevated-plus maze followed by the forced swimming test (both performed in the sixth week), Morris water maze (seventh and eighth weeks), rotarod followed by open field and the sucrose preference test (all in week 10), and the skilled paw reaching test (week 11). The minimum interval between two consecutive procedures was 2 days. All animals were subjected to the same sequence of behavioral test and sacrificed 12 weeks after surgery. Rats were deeply anesthetized with pentobarbital and were transcardially perfused with 4% paraformaldehyde (PFA) in PBS. The brain was then removed surgically for immunohistochemistry (IHC) analysis.

### 6-OHDA or AAV α-syn injection in the substantia nigra

Under ketamine and medetomidine (75 mg/kg: 0.5 mg/kg, i.p.) anesthesia, the animals were placed on a stereotaxic frame. Animals were pre-treated with desipramine (25 mg/kg) 30 min prior to 6-OHDA injection in order to protect noradrenergic neurons. Bilateral injections of 8 or 6 μg of 6-OHDA (dissolved in 2 μl of 0.2% ascorbic acid saline solution) were performed in the SNpc [coordinates with respect to bregma: A −5.3; L ±1.8; V −7.4 mm according to the stereotaxic atlas of Paxinos and Watson (1998)] by means of a 10 μl Hamilton syringe. A different group of animals was also injected stereotaxically using the same protocol with 2 μl of AAV serotype 2 (AAV2) to selectively overexpress human α-syn. Control (sham) animals received the same volume of vehicle, following the same procedure. The solutions were infused at a rate of 0.5 μl/min and after the injection the needle was left in place for an additional 2 min period before it was slowly retracted.

### Implantation of paraquat (PQ) mini-osmotic pumps

Under anesthesia with ketamine and medetomidine animals were implanted with Alzet mini-osmotic pumps (Model 2004, Durect™) delivering for 28 days at a rate of 0.7 mg/day PQ (Sigma). Implants were placed in the subscapular region.

### Construction of human α-synuclein delivery vector U6-CMV-EGFP/pAAV (AAV-α-syn) and preparation of rAAV2 containing a-syn

U6 promoter-driven α-syn expression system was established in AAV2 vector, with EGFP expression separately controlled by a CMV promoter as a marker for the transduction efficiency. The plasmid DNA vector only or AAV2-α-syn were co-transfected with plasmids pHelper and pAAV-RC to HEK293 AAV cells using a standard calcium phosphate method. At 72 h post-transfection, the cells were harvested and crude rAAV vector solutions were obtained by repeated freeze/thaw cycles. The cleared crude lysate was applied on a heparin column. After the total lysate passed through the column, the matrix was washed twice with 25 ml of PBS with low NaCl (pH7.4, 0.1 M NaCl) and the virus eluted with 15 ml of PBS with high NaCl (PBS; pH 7.4, 0.4 M NaCl). The elute was concentrated to about 1 ml using a Millipore Biomax-100K NMWL filter device (UFV2BHK40) by centrifugation at 4000 rpm, 15–40 min. To adjust the NaCl concentration to physiological levels, the filter device was refilled with PBS (pH 7.4) and the virus concentrated to 250–300 μl. After removal of the virus-containing solution, the membrane of the filter device was washed three times with PBS (pH 7.4) that was added to the main part of the recombinant AAV2. The fractions containing high-titer rAAV vectors were collected and used for injection into animals. The number of rAAV genome copies was semi-quantified by PCR within the CMV promoter region using primers 5′-GACGTCAATAATGACGTATG-3′ and 5′-GGTAATAGCGATGACTAATACG-3′. The final titers were 6.4 × 10^11^ genomes/μl (rAAV2-vector) and 5.5 × 10^11^ genomes/μl (rAAV2-α-syn). Each animal received 11 × 10^11^ genomes of the respective rAAV-vector.

### Behavioral assessment

#### Elevated plus maze

Anxiety-like behavior was measured in the elevated plus maze as previously described (Pego et al., 2008). The apparatus comprised two open arms (50.8 × 12 cm) and two closed arms (50.8 × 12 × 40.6 cm) that extended form a common central platform (10 × 10 cm), raised 72.4 cm above the floor. Each animal was individually placed in the central platform facing the open arm and allowed to explore the maze freely for 5 min. After each session the apparatus was cleaned with 10% ethanol. The test was recorded via a video camera and the behavioral analysis was carried out using The Observer Basic 3.0 software. The behavioral parameters recorded were: number of entries into the open and closed arms; and, percentage of time spent in the open and closed arms, and at the arms intersection. Percentage of time spent in the open arms was used as an index of anxiety-like behavior, whereas the number of entries in closed arms was taken as a measure of locomotor activity, which is considered more valid than total number of arms entries (Wall and Messier, 2001).

#### Forced swim test

The forced swim test was used to evaluate depressive-like behavior, as previously described (Leite-Almeida et al., 2009). Rats were placed in a cylinder filled with water (24°C), for a period of 5 min. The test was recorded via a video camera and latency to stop and immobility time were determined. Depressive-like state was defined as an increase in time of immobility and decrease in latency to immobility.

#### Morris water maze

Cognitive function was assessed in the Morris Water Maze test, which is composed by working memory, spatial reference memory, and reversal learning tasks. The tasks were performed essentially as described by Cerqueira et al. (2007). Briefly, the tests were made in a circular black tank (170 cm diameter), filled with water at 24°C to a depth of 31 cm in a dimly illuminated room with extrinsic spatial clues on the walls. The tank was divided in imaginary quadrants and had an invisible platform placed within one of them. Data were collected using a video camera fixed on the ceiling, above the center of the tank and connected to a video-tracking system (View Track v.2.6., ViewPoint Life Sciences Inc.). The position of the platform varied according to each task. In each day of test, animals performed four trials to find the platform. At the beginning of each trial, animals were placed in the periphery of the selected quadrant facing the wall of the maze. Trials were automatically ended once the animals reached the platform; if the animal failed to find the platform in 120 s, it was gently guided to it. Between each trial the animals were allowed to rest in the platform for 20 s. Time to reach the platform and the distance traveled were automatically recorded. The working memory task measures the capacity to learn the location of the platform and to retain this information during four consecutive trials. This task was conducted during 4 days, throughout which the platform was hidden in a different position each day. The last day of the working memory task corresponded to the first day of the spatial reference memory task, which was designed to evaluate the ability to learn the position of the platform during three consecutive days. The platform remained in the same position (“old” quadrant) throughout the 3 days. The reversal learning task was performed on the seventh day, assessing for cognitive flexibility. During the task, the platform was positioned on the opposite quadrant (“new” quadrant) of the last 3 days and animals were similarly tested during four trials. On this last day of the test a fifth trial was added, the probe trial, in which the platform was removed from the tank and each animal was tested for a 120 s period. The distance swum in each quadrant was recorded in all five trials.

#### Rotarod

The motor performance was performed at 8 weeks post-surgery using the rotarod equipment (3376O4R, TSE Systems) as previously described (Monville et al., 2006). The unit consists of a rotating spindle, a power source for turning the spindle and grids beneath the rotating roller where the rat can fall without injury. All animals were pre-trained on the rotarod apparatus in order to reach a stable performance. The training consisted of four sessions on 3 consecutive days, under an accelerating protocol starting at 4 rpm and reaching 40 rpm in 5 min; each session included three separate trials, with at least 20 min of rest between trials. At the fourth day the final test was performed under the same accelerating protocol and the latency to fall was recorded.

#### Sucrose preference test

Anhedonia was assessed using the sucrose preference test previously described by Carvalho et al. (2013). The test consisted of depriving animals food and water for 18 h and afterwards presenting two preweighted (separate) bottles, one containing 3% sucrose solution and a second containing tap water, during 1 h. Sucrose preference was calculated according to the formula: sucrose preference = (sucrose intake/(sucrose intake + water intake)) × 100. Reduced sucrose preference is suggestive of anhedonia and thus of depression-like behavior. In addition, we also analyzed the total sucrose and water consumption to compare general fluid intake between the groups.

#### Open field

The open field was used to assess locomotor behavior as previously described (Leite-Almeida et al., 2009). The rat was placed in the center of a square (43.2 cm × 43.2 cm) arena (ENV-515, Med Associates Inc.) and allowed to explore the arena for 5 min. With the help of the tracking software (SOF-811, Med Associates Inc.) total distance traveled, time in rest, number of rearings and ambulatory episodes were recorded. The arena was cleaned with 10% ethanol between each animal.

#### Skilled paw reaching test

Skilled paw reaching was assessed using a double staircase box (80300, Campden Instruments Ltd.); shape and dimensions were similar to the device described by Montoya et al. (1991). The apparatus was developed to assess the independent forelimb use in skilled reaching and grasping task. Briefly, the apparatus consists of a clear chamber with a hinged lid. A narrow compartment, with a central platform running along its length, is connected to this chamber. A removable double staircase with 7 steps on each side can be inserted in the space between the platform and the box walls. Three pellets were placed into each well of the double staircase apparatus. In the first 2 days, the rats were familiarized with the test, with the pellets being available for 5 and 10 min, respectively. In the test session, the animals were placed inside the box and had 15 min to reach, retrieve, and eat the food pellets placed on the steps. All sessions were performed at the same time of the day and with food restricted animals. After the period test, the animals were removed from the box and the remaining (left over) pellets counted.

### Immunohistochemistry

IHC was carried out in free-floating mesencephalic axial frozen sections (35 μm). The chromogenic IHC assay was used to detect DAergic neurons. Nonspecific antibody binding was blocked by incubation for 60 min in PBS with 10% FCS containing 0.1% Triton X-100. Endogenous peroxidase activity was quenched by 10 min incubation in 1% H_2_O_2_. Sections were incubated with mouse anti-tyrosine hydroxylase (TH) (1:1000; Transduction Laboratories) diluted in PBS with 1% FCS. Incubations with the primary antibodies were performed overnight at 4°C. Sections were then incubated for 1 h at room temperature with biotinylated secondary antibody, goat anti-mouse IgG (1:200; Vector Laboratories), diluted in PBS. After that sections were incubated with avidin peroxidase (1:1000 in PBS; Vector Laboratories) for 50 min at room temperature. The reaction product was visualized using diaminobenzidine (DAB, Sigma) in TBS with 0.0024% H_2_O_2_.

### Quantitative analysis of TH positive cells

The immunohistochemically processed sections were used to evaluate the total number of TH immunoreactive cells within the SNpc. On mesencephalic sections, the region comprising the TH positive cells, correspondent to SNpc, was delineated and the total number of TH positive cells was counted in the full extent of the structure per section. Every sixth section covering the entire extent of the SNpc was included in the counting procedure. The following formula was used to estimate the number of TH neurons: *N* = *V*_(*SNpc*)_ (Σ*Q*/Σ*V*_(sect)_); where *N* is the estimation of cell number, *V* is the total volume of SNpc, Σ*Q* is the total number of cells counted in the sections, and Σ*V*_(sect)_ is the total volume of counted sections.

### Statistical analysis

All data sets were tested for normality distribution with the Shapiro–Wilk test prior to any statistical analysis. Data are expressed as mean ± standard error of mean (s.e.m.). Statistical analysis was performed using Student *t*-test, Mann–Whitney nonparametric test, one-way or repeated measures analysis of variance (ANOVA), as appropriate. Differences between groups were further analyzed by Dunnett's or Bonferroni's post tests. *p*-value of 0.05 was considered statistically significant. All statistical procedures were performed using GraphPad Prism, version 4 (GraphPad Software Inc., San Diego, CA, USA).

## Results

### Assessment of the extent of the lesion

**Figure 1:**
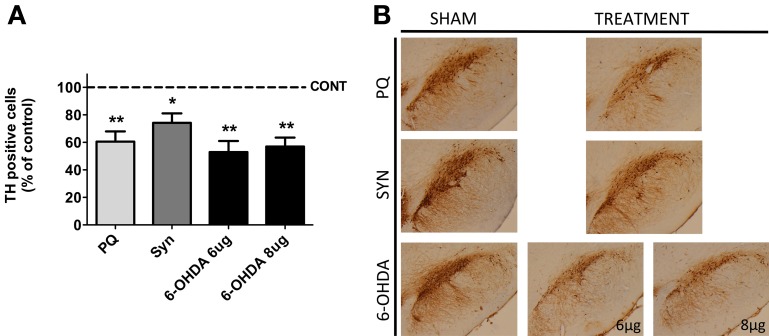
**Dopaminergic cell loss induced by PQ, α-syn overexpression and 6-OHDA.** Eleven weeks after lesion, the animals were sacrificed and the substantia nigra region was processed for tyrosine hydroxylase (TH) immunohistochemistry **(A)**. Representative photomicrographs of midbrain sections immunostained for TH are presented in **(B)**. Data are expressed as the percentage of control animals and represent the mean ± s.e.m. of at least five animals per group. ^*^P < 0.05 and ^**^P < 0.01 compared to control.

Histological analysis of TH positive cells in the SNpc revealed a significant loss of DAergic neurons in all the animal models (Figure 1). The highest difference [*F*_(2.11)_ = 15.32; *p* = 0.0007] was observed in 6-OHDA model, when compared with sham rats, with *post-hoc* analysis showing a marked decrease in the number of TH positive cells in the SNpc of animals treated with both 8 and 6 μg of 6-OHDA (43.04 ± 4.673% and 47.08 ± 4.819%, respectively). In the PQ-exposed rats a 39% reduction of nigral DAergic neurons was also observed (*U* = 0.0; *p* = 0.0022). The less severe lesion (21%) was induced by the overexpression of α-syn (*U* = 6.0; *p* = 0.02).

### Motor performance

**Figure 2:**
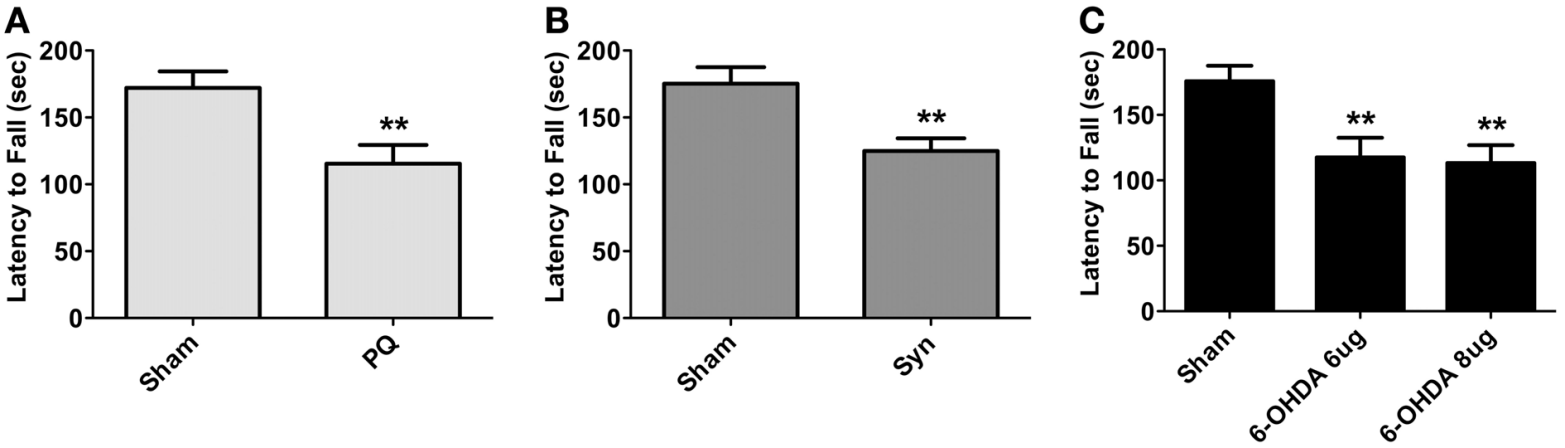
**Motor performance of PQ (A), α-syn (B) or 6-OHDA (C) treated animals measured as latency to fall in accelerating rotarod test.** Data are presented as mean ± s.e.m. ^**^p < 0.01.

General motor performance was assessed at 8 weeks after surgeries, using the accelerating rotarod test. Animals in all PD models displayed significant reductions in the latency to fall [*t* = 3.498 for PQ; U = 172.0 for α-syn; *F*_(2.41)_ = 6.726 for 6-OHDA; all *p* < 0.01] (Figure 2).

**Figure 3:**
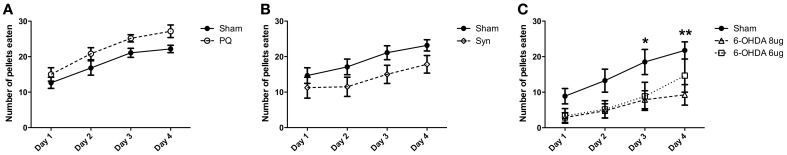
**Effects of PQ delivery (A), α-syn overexpression (B) or 6-OHDA injection (C) on paw reaching performance.** Performance of rats in the paw reaching test is expressed as mean ± s.e.m. number of eaten pellets. ^*^p < 0.05 and ^**^p < 0.01.

The skilled paw reaching test was use to assess forelimb use; this test was performed 10 weeks post-lesion and the number of pellets eaten was evaluated. Regarding the PQ model, ANOVA showed an effect for the factor time [*F*_(3.45)_ = 29.17; *p* < 0.001], but no effect for the factor lesion [*F*_(1.45)_ = 4.526; *p* = 0.0504], as well as no interaction between these factors [*F*_(3.45)_ = 0.3662; *p* = 0.777] (Figure 3A). In the α-syn model, a significant effect of time [*F*_(3.84)_ = 21.66; *p* < 0.0001] and lesion [*F*_(1.84)_ = 2.871; *p* = 0.1013] was verified, while no interaction was detected between these two factors [*F*_(3.84)_ = 0.6312; *p* = 0.5969] (Figure 3B). Similar results were obtained in rats injected with 6-OHDA, with a significant effect of time [*F*_(3.57)_ = 23.42; *p* < 0.001] and lesion [*F*_(2.57)_ = 4.363; *p* = 0.0276], without interaction between the factors [*F*_(6.57)_ = 1.136; *p* = 0.3533] (Figure 3C). *Post-hoc* testing revealed decreased success in the number of eaten pellets in rats treated with 6-OHDA high dose in comparison to sham, at day 3 and 4 (*p* < 0.05 and *p* < 0.01, respectively).

### Exploratory and locomotor activity

**Figure 4:**
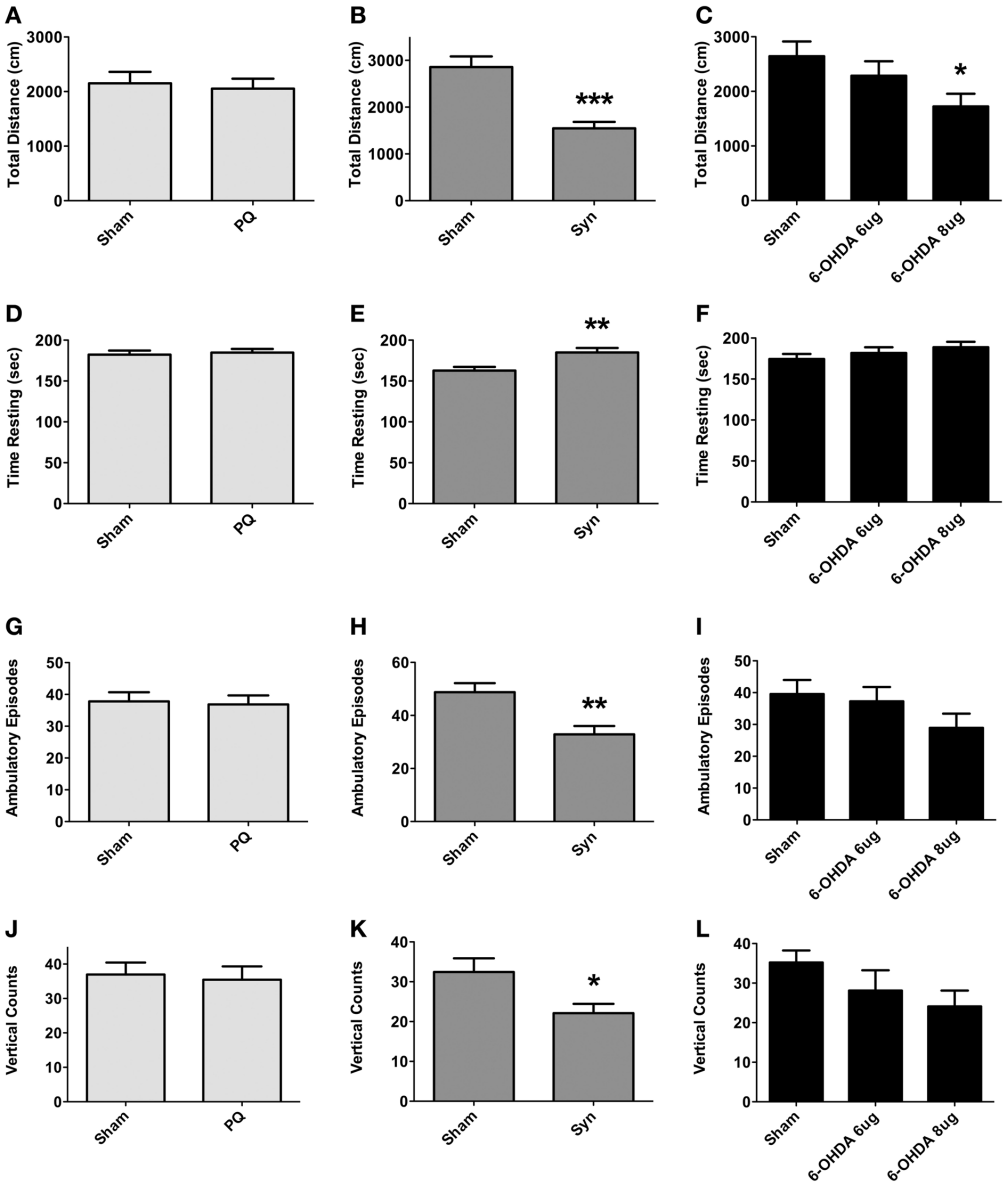
**Locomotory and exploratory activity.** Differences in total distance traveled **(A–C)**, time in rest **(D–F)**, number of ambulatory episodes **(G–I)** and number of rearings **(J–L)**. Data expressed as mean ± s.e.m. ^*^p < 0.05, ^**^p < 0.01, and ^***^p < 0.001.

The open field was the test was to used to assess exploratory activity; the test was carried out at 9 weeks post-surgery. Exploratory activity did not differ between sham and PQ rats, with no differences found in the total distance traveled (*U* = 548.398; *p* = 0.6334), number of ambulatory episodes (*U* = 200.0; *p* = 0.5008), and number of rearings (*t* = 0.2845; *p* = 0.7775) (Figures 4A,D,G,J). Overexpression of α-syn resulted in a decrease in the total distance traveled (*U* = 113.0; *p* < 0.0001) accompanied by an increase in the rest time. Significant differences were also observed regarding the number of ambulatory episodes, with α-syn rats presenting lower number when compared with sham animals (*t* = 3.242; *p* = 0.0020), and the number of rearings was also decreased (*U* = 57.5; *p* = 0.0333) (Figures 4B,E,H,K). Finally, an overall effect was found on the total distance traveled in 6-OHDA lesioned rats [*F*_(2.46)_ = 3.34; *p* = 0.0442]. A *post-hoc* comparison revealed that animals treated with 6-OHDA 8 μg traveled significantly less than sham rats. In contrast, 6-OHDA 6 μg rats traveled a total distance that did not differ from that observed in the sham group. No differences were observed in ambulatory episodes [*F*_(2.48)_ = 1.595; *p* = 0.2134] and in number of rearings [*F*_(2.47)_ = 2.061; *p* = 0.1387], in both 6-OHDA doses (Figures 4C,F,I,L).

### Anxiety-like behavior

**Figure 5:**
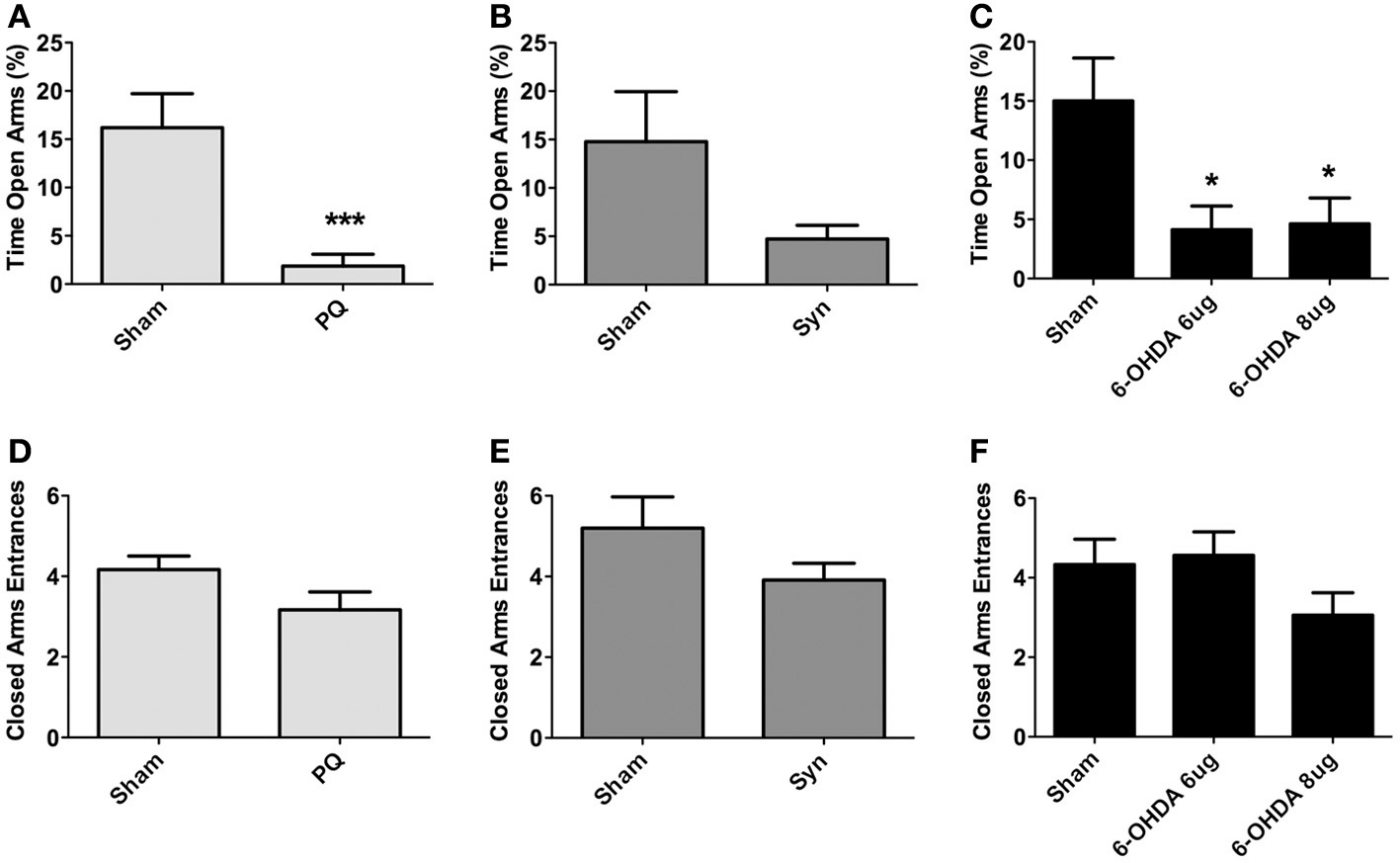
**Anxiety-like behavior in the elevated-plus maze.** Data on percentage of time in open arms **(A–C)**, and number of entries in closed arms **(D–F)**. Data expressed as mean ± s.e.m. ^*^p < 0.05 and ^***^p < 0.001.

The elevated plus maze was used to assess for anxious-like behavior. Data showed that both PQ and 6-OHDA lesions affected anxiety-like behavior. Specifically, regarding the percentage of time spent on the open arms, treated animals from these groups spent less time in the open arms when compared to sham animals [*U* = 24.50, *p* < 0.0001 for PQ; *F*_(2.38)_ = 5.203, *p* = 0.0101 for 6-OHDA] (Figures 5A,C). The same tendency was verified for α-syn rats, but no statistical significant differences were found (*U* = 222.0; *p* = 0.1882) (Figure 5B). No statistically significant differences were found in the number of entries on the closed arms in any of the lesion models [*U* = 150.0, *p* = 0.0914 for PQ; *U* =268.0, *p* = 0.1945 for α-syn; *F*_(2.48)_ = 1.799, *p* = 0.1765 for 6-OHDA] (Figures 5D–F).

### Depressive-like behavior

**Figure 6:**
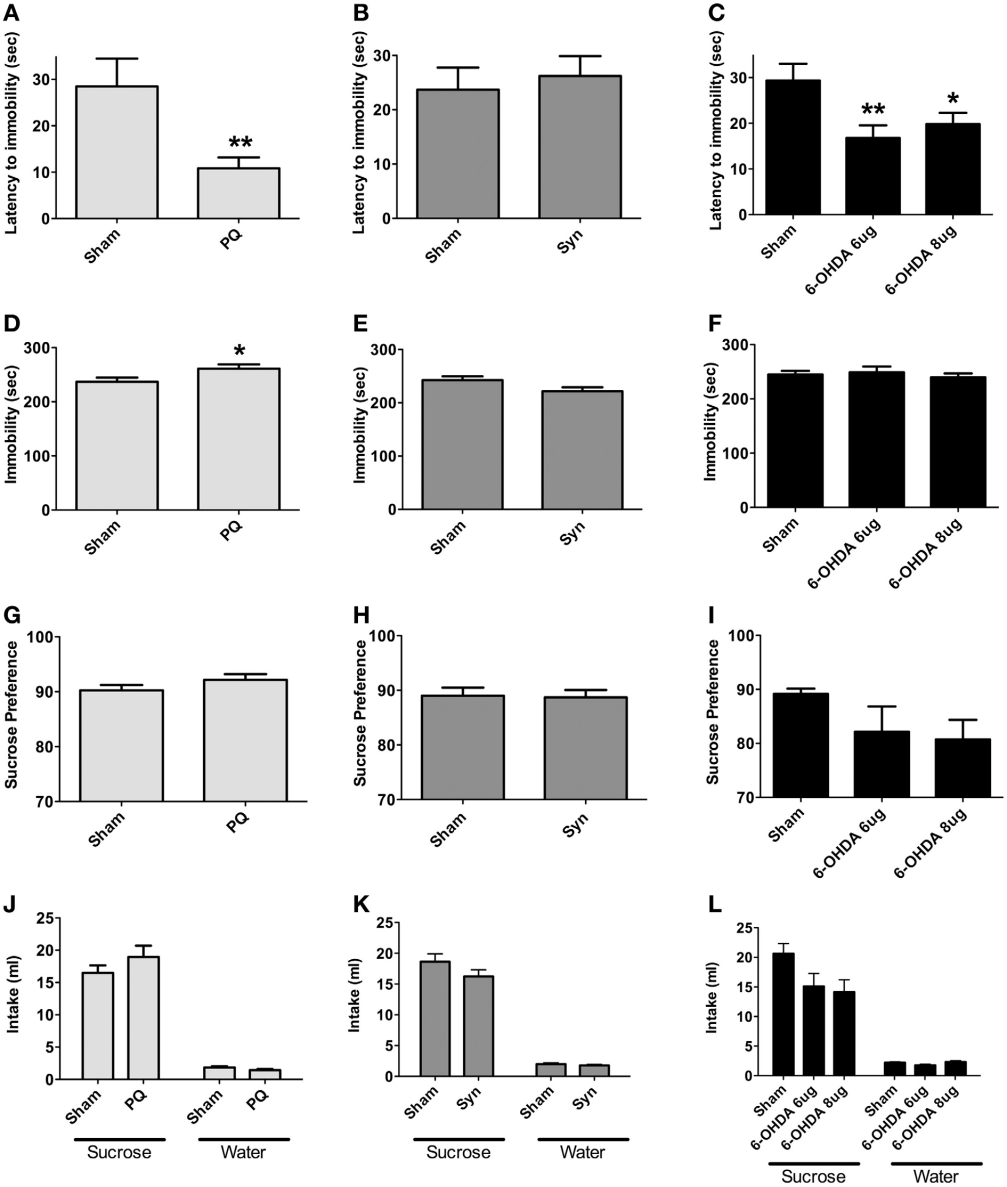
**Forced swimming test (A–F) for depressive-like behavior.** Data regarding latency to immobility **(A–C)** and immobility time **(D–F)**. Presence of anhedonia, assessed through the sucrose preference test **(G–I)**. Total sucrose and water consumption during the sucrose preference test **(J–L)**. Data expressed as mean ± s.e.m. ^*^p < 0.05 and ^**^p < 0.01.

Forced swimming test was used to assess depressive-like behavior; in addition, sucrose preference test was conducted to evaluate for anhedonia. PQ delivery and bilateral 6-OHDA injection in the SNpc significantly affected behavioral response in latency to stop [*U* = 70.0, *p* = 0.0028 and *F*_(2.34)_ = 6.658, *p* = 0.0036, respectively] (Figures 6A,C). *Post-hoc* tests revealed that latency to stop time was shorter in 6-OHDA animals treated with either of the 6-OHDA doses, when compared to the sham rats, although this difference was more pronounced with the lower dose (*p* < 0.05 for 6-OHDA 8 μg; *p* < 0.005 for 6-OHDA 6 μg). Total immobility time was, however, only affected in the PQ model (*U* = 121.0, *p* = 0.0290) and not in the 6-OHDA model [*F*_(2.47)_ = 0.3016, *p* = 0.741] (Figures 6D,F). In α-syn model no differences were found (latency to stop: *U* = 356.5, *p* = 0.7283; immobility: *U* = 290.0, *p* = 0.1128) (Figures 6B,E).

Regarding sucrose preference no differences were observed in all the PD models studied [*U* = 141.0, *p* = 0.1162 for PQ; *U* = 385.0, *p* = 0.9613 for α-syn; *F*_(2.44)_ = 1.786, *p* = 0.1795 for 6-OHDA] (Figures 6G–I). Nevertheless, in animals injected with 6-OHDA (at both doses) a trend for decreased preference for sucrose was observed.

In addition, the different lesions did not significantly affect the total amounts of sucrose and water intake (Figures 6J–L).

### Cognitive performance

**Figure 7:**
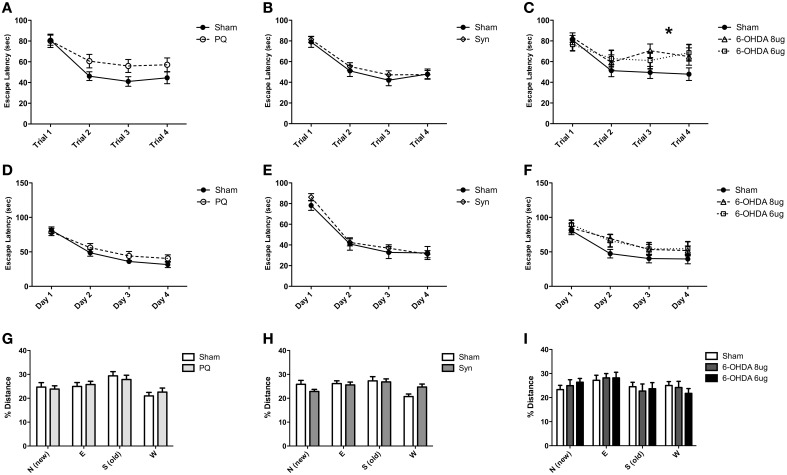
**Cognitive behaviors.** Animals were challenged with three tasks that differed in their cognitive requirements. Working memory performances of sham and injured animals are compared, PQ **(A)**, α-syn **(B)** and 6-OHDA **(C)** animals. Similarly, spatial reference memory performance is plotted in graphs **(D–F)** and reversal learn task performances **(G–I)**. The four quadrants of the pool are assigned with letters N, E, S, W; the actual and the former platform positions are, respectively, N and S. ^*^p < 0.05.

The Morris water maze was performed to evaluate cognitive functions. The protocol was divided in three different tasks to investigate both working and spatial reference memories and cognitive flexibility. No interaction between lesion and time were found in PQ and α-syn models in any of the tasks (all *F*-values > 0.2635, all *p*-values > 0.0714) (Figures 7A,B,D,E,G,H). However, in the 6-OHDA model, escape latency analysis of working memory revealed a significant interaction between lesion and daily performance [*F*_(6.144)_ = 2.25; *p* = 0.0417] and a significant effect of time [*F*_(3.144)_ = 18.95; *p* < 0.001] (Figure 7C). *Post-hoc* comparison revealed an improvement in daily performance in the sham group (all *p*-values < 0.001, for each day as compared to the first day), which was not observed in 6-OHDA rats. In high dose of 6-OHDA the improvements were observed between the first and second, and first and fourth days (*p* < 0.001 and *p* < 0.01, respectively) whereas there was no difference between first and third day. Rats injected with low 6-OHDA dose only improved the performance between first and third day (*p* < 0.05). In the other tasks the escape latency and distance swam did not differ between the groups (all *F*-values > 0.4394, all *p*-values > 0.743) (Figures 7F,I).

## Discussion

The present work confirmed that all three animal models of PD trigger the characteristic motor symptoms of the disease, as a consequence of reduced number of TH-positive neurons in the SNpc. Furthermore, on the latter parameter, we found a range of variation from 20 to almost 50% in the reduction of TH-positive cells, which is within the ranges of variance observed in other studies using similar animals models (Kirik et al., 2002; Pioli et al., 2004; Cristovao et al., 2009, 2012; Decressac et al., 2012b). However, we failed to find a significant correlation between the DAergic neuronal degeneration and the severity of the motor deficits; therefore, we cannot make an association between each model and a stage of the disease progression, as have been done in other studies.

The main goal of this study was, however, to characterize the NMS in each of these animal models. First, it is relevant to highlight, here animal models that triggered DAergic degeneration on both hemispheres were chosen, which is of importance to the emergence of the full spectrum of behavioral symptoms of the disease. Briefly, from analysis of an extensive battery of behavioral tests, the PQ, the 6-OHDA and the α-syn models of PD were shown to have distinctive patterns of NMS impairments.

### Effects of PQ induced lesion

The use of the herbicide PQ to study PD and NMS is still emerging. In contrast, the study of motor deficits in this model has already been done both in rats (Somayajulu-Nitu et al., 2009) and mice (Litteljohn et al., 2008). Herein we confirmed the existence of deficits in the motor performance of PQ-exposed rats; however, impairments were visible on the rotarod, but not in the skilled paw-reaching test. These results suggest that, in spite of the global motor coordination deficits produced by PQ, these animals presented normal fine motor control. Furthermore, the present study also indicated non-motor alterations in this model of PD. In particular, PQ treated rats spent less time in open arms in the elevated plus maze; this difference cannot be ascribed to motor deficits as the number of closed arms entries was similar in PQ and sham rats. Therefore, and in contrast to previous observations in mice (Litteljohn et al., 2008), these findings demonstrate that PQ induces anxious-like symptoms in rats. As to depressive-like behavior, we found that PQ treated rats displayed increased immobility, as well as a decreased latency to stop in forced swimming test, but no changes in anhedonic behavior. As the latter test is less dependent on locomotion, the former changes must be regarded with caution. Finally, no major cognitive deficits were found in PQ treated rats. The lack of deficits in these behavioral domains merits to be highlighted as it is known that PQ not only affects the nigrostriatal pathway but also accumulates in the cortex and in the hippocampus (Prasad et al., 2009), with one study revealing the ability of PQ to exacerbate cognitive impairments in an Alzheimer's disease model (Chen et al., 2012). We can hypothesize that our low-dose chronic PQ infusion regimen was not enough to accumulate in these brain regions and to cause significant deficits in the tasks we assessed, since the classic PQ rodents models of PD used in previous studies are achieved by exposure to higher doses of PQ.

### Effects of α-syn overexpression

Injection of viral vectors in the SNpc, to promote the overexpression of wt or mutated human α-syn, has recently proved to be a promising model of PD. Confirming previous studies (Decressac et al., 2012b), the animals injected with AAV2-α-syn vector presented a reduction in locomotor activity measured in the rotarod, even though it was associated with a less striking neuronal loss in the SNpc. Interestingly, in comparison with the other two models herein studied, this approach was the poorest regarding NMS. In fact, only a decreased exploratory activity in the open field test was observed. Otherwise, no significant changes were observed in anxiety- and depressive-like behaviors, and their learning and memory abilities seemed to be unaffected by the overexpression of α-syn (a similar phenotype to the one observed in PQ model). These results seem to be contradictory with previous reports using α-syn models, namely at the cognitive performance level (Freichel et al., 2007; Masliah et al., 2011; Magen et al., 2012). It is, however, important to note that previous studies assessing NMS in α-syn models have been performed in transgenic mice in which α-syn aggregates are distributed through the cortex and hippocampus similar to what has been described in PD with dementia (Churchyard and Lees, 1997; Hurtig et al., 2000).

### Effects of 6-OHDA lesion

The most widely used model of PD consists in the injection of 6-OHDA into one of the following three target sites: SNpc, medial forebrain bundle or the caudate-putamen. In spite of being the most scrutinized, including NMS, there is still a lack of studies using the bilateral 6-OHDA lesion model. Here, rats injected with two doses of 6-OHDA, bilaterally into the SNpc, displayed motor deficits accompanied by several NMS; the latter including increased anxious and depressive-like behaviors, and working memory deficits. Surprisingly, no differences were observed between the two doses of 6-OHDA, suggesting that lower dose is sufficient to produce all the effects observed, being in accordance with the similar impact that both doses had on DAergic neurons degeneration.

More specifically, the motor performance, assessed through the rotarod and skilled paw-reaching test, was clearly impaired. Motor abnormalities were also observed in the open field test, where 6-OHDA animals treated with higher dose displayed a shorter travel distance. In the elevated plus maze test, 6-OHDA treated animals showed a significant decrease in time spent in the open arms, while no differences in closed arms were observed, thus suggesting an anxious-like state in these animals. The anxious phenotype is in line with observations showing that infusion of 6-OHDA in the striatum also results in an increase in anxiety-like behavior (Tadaiesky et al., 2008). Regarding mood behavior, previous studies reported an increase in depressive-like behavior after bilateral 6-OHDA into the SNpc or striatum, assessed both by sucrose preference test and forced swimming test (Branchi et al., 2008; Santiago et al., 2010). Although, no significant effect was found in the sucrose preference test, the tendency to an anhedonic behavior in lesioned rats, herein observed, was reinforced by a shorter latency to immobility in forced swimming test. This may suggest a predisposition to a depressive phenotype induced by bilateral 6-OHDA lesion. Noticeably, this was the only model presenting cognitive deficits as measured by differences in working memory performance, a task highly dependent on DA levels in the prefrontal cortex (D'Hooge and De Deyn, 2001; Cerqueira et al., 2007).

### Concluding remarks

In conclusion, here the data demonstrates that all the models included in this study present the crucial requirements for a model of PD, namely a selective loss of DAergic neurons, together with detectable motor deficits. Nonetheless, in addition to the classical features of PD, these models displayed different patterns of NMS symptoms. Overall, the α-syn model proved to be useful to study changes in exploratory activity, while the PQ model offered a valuable approach to investigate neuropsychiatric dysfunction, specifically alterations in depression and anxiety-like behavior. Finally, the 6-OHDA model displayed the broader spectrum of NMS and thus might be of use to study in combination emotional and cognitive deficits associated to PD. In future studies, the intrinsic differences of these toxin and genetic models can be relevant to unravel the mechanism underlying NMS, as well as to screen drugs for symptomatic treatment of the disease.

## Authors' contributions

F. L. Campos performed surgeries, behavioral assessment, histological analysis, treatment protocols, data analysis and interpretation, and drafted the manuscript. M. M. Carvalho helped in surgical procedures and behavioral analysis. A. C. Cristovão helped in AAV-vector production and in revision of manuscript. G. Je produced the AAV-vector. G. Baltazar, A. J. Salgado, and Y. S. Kim helped in revision of the manuscript. N. Sousa contributed for data interpretation and for drafting, revision, and approval of the manuscript. All authors read and approved the final manuscript.

### Conflict of interest statement

The authors declare that the research was conducted in the absence of any commercial or financial relationships that could be construed as a potential conflict of interest.
